# Intrahepatic cholestasis of pregnancy: Relationship between bile acid levels and maternal and fetal complications

**DOI:** 10.4274/tjod.28000

**Published:** 2014-09-15

**Authors:** Bilge Çetinkaya Demir, Esra Şahin Güneş, Mehmet Aral Atalay

**Affiliations:** 1 Uludağ University Faculty of Medicine, Department of Obstetrics and Gynecology, Bursa, Turkey

**Keywords:** Intrahepatic cholestasis of pregnancy, preterm birth, perinatal morbidity, serum bile acid levels

## Abstract

**Objective:**

Intrahepatic cholestasis of pregnancy (ICP) complicates pregnancies which is characterized by elevated serum bile acid levels. ICP increases maternal and fetal morbidities. This study was designed to determine the association of maternal and fetal complications and serum bile acid levels.

**Material and method:**

Maternal and fetal characteristics were analyzed from the medical records of 61 patients who gave birth following a pregnancy complicated with ICP between 2009 and 2013.

**Results:**

Eighty seven percent of 61 cases were singletons, and 13% of them were twins. Mean SBA level was 36 μmol/L. Preterm birth rate among singletons and twin pregnancies were 24.5% and 62.5%, respectively. Mean SBA level in preterm birth group was statistically higher with respect to the term birth group (100.8 μmol/L and 25.61 μmol/L, respectively; p=0.001). No perinatal mortality associated with ICP was detected in the study group.

**Conclusion:**

Pregnant women with the ICP compose high-risk group in regard to fetal and maternal risks. Close follow-up of these patients is required due to increased risks such as preterm delivery, meconium staining and fetal death.

## INTRODUCTION

Intrahepatic cholestasis of pregnancy (ICH) is a disease characterized with severe pruritus and increased levels of serum bile acids (SBA), seen in second and third trimesters of pregnancy. It is reported to complicate 0.1-15% of pregnancies in different series and is the most common pregnancy associated hepatic disorder^(1,2,3)^. Major symptom is pruritus of all body especially located to palms and soles and which increases at nights^(4)^. The prevalence of ICH is significantly increased in twin pregnancies^(5)^. Since there are studies trying to explain etiologic factors (such as hormonal, genetic and inflammatory) causing the increased levels of maternal SBA levels, the etiology is not clear yet.

There is increased level of liver enzymes but diagnosis should be based on elevated levels of SBA. Other laboratory findings of cholestasis also accompany this increased level of bile acids. The differential diagnosis of viral hepatitis should be made in patients with severely-increased levels of aminotransferases^(6)^. The diagnosis of ICH should be based on laboratory findings of liver dysfunction in patients with severe pruritus^(7)^. The pruritus and the deteriorated liver enzymes typically returns normal levels 4 weeks after delivery^(8)^.

Ursodeoxycholic acid (UDCA) is used in the treatment of IHC^(9)^. It is found to be more effective on maternal symptom, decreasing serum bile acid and liver enzymes levels than cholestyramine and dexamethasone^(9,10)^. UDCA is also found to regulate the plasental bile acid transport^(10,11)^.

In contrast to benign maternal course in ICH, the fetuses face with complications such as preterm labor, meconium stained amniotic fluid and intrauterine fetal demise^(12)^. There is no ideal fetal follow up protocol for ICH cases. Fetal complications cannot be anticipated by ultrasonography or cardiotocography. In this retrospective study we aimed to evaluate perinatal outcomes of ICH cases in our clinic and to evaluate the relation of ICH with preterm labor.

## MATERIAL AND METHODS

### Study Participants

Patients with ICP were selected from medical records of our perinatology clinic enclosing the interval between June 2009 and June 2013. Elevated serum bile acid levels above 10 μmol/L in patients who are in between 24^th^ and 40^th^ gestational weeks of pregnancy, having complaints of pruritus, and/or elevated liver enzymes were diagnosed as ICP. Patients with active viral hepatitis, dermatologic causes of pruritus, gall bladder and liver disorders, and preeclampsia were excluded from the study. Patients who are diagnosed as ICP were followed-up with serial ultrasonographic examinations and fetal biophysical profile weekly for the assessment of fetal well-being. Ursodeoxycholic acid was administered in 10 to 15 mg/kg/day in divided dosages according to the severity of symptoms and serum levels of liver enzymes. During follow-up, labor was induced in presence of non-reassuring non-stress test and/or an increase in serum levels of the liver enzymes above 10 times of the normal values.

Age, gravidity, parity, gestational age at diagnosis, body mass index (BMI), highest SBA value, liver aminotransferase level, severity of symptoms, delivery time, mode of delivery of the patients and APGAR score at 5^th^ minute, birth weights of the newborns were recorded. Deliveries before 37^th^ gestational week for the singletons, and deliveries before 36^th^ gestational week for the twin pregnancies were defined as preterm delivery. Additionally, full-term and preterm deliveries were divided into iatrogenic and spontaneous delivery groups.

### Statistical Analyses

Statistical analyses were conducted by Statistical Package for the Social Sciences (SPSS) 22.0 (Chicago, IL.). Student’s t-test and Mann-Whitney U-test were used in the comparisons of continuous variables, which are distributed normally and abnormally, respectively. Spearman’s rank correlation coefficient was calculated for the definition of the strength of possible associations. Pearson’s chi-squared test was used in the comparisons of groups consisting categorical variables. A p-value <0.05 was accepted as the statistical significance.

Sixty-two patients were diagnosed as ICP at study period. One patient was excluded from the study because she had triplet pregnancy. Eight (13%) of the 61 patients had twin pregnancies, while 53 (87%) patients were singletons. Median age of the study participants was 28 (20-43) years. Median gestational age at the diagnosis was 34 weeks (26-36 weeks) in patients with twin pregnancies, and 37 weeks (25-39 weeks) for singletons. At the time of diagnosis, median SBA level was 36µmol/L, median alanine aminotransferase (ALT) and aspartate aminotransferase (AST) levels were 191 IU/L and 109 IU/L, respectively (Table 1).

Delivery was achieved by cesarean section in 42.6% of the participants. The ratio of cesarean section was %60 for singletons, and %62.5 for twin pregnancies. The most common indication for cesarean section was fetal distress with a rate of %46. In our follow-up, there were not perinatal or maternal deaths. Only one of the patients needed blood transfusion because of peripartum hemorrhage as a complication of delivery.

Twenty-nine patients had spontaneous labor and 32 patients (52%) were induced for labor because of the fetal and maternal reasons. Mean gestation age at delivery was 35 weeks for spontaneous delivery group and 37 wks for induction group. Mean SBA value was 26.9 µmol/L in the induction group and it was 34.5 µmol/L in spontaneous delivery group but the difference was not statistically significant (p=0.094) (Table 2). Number of the patients with iatrogenic preterm delivery was less than iatrogenic term delivery group (4 vs. 28 patients) (p=0.001) (Table 3).

Sixty two point five percent (n=5) of twin pregnancies and 24.5% (n=13) of singleton pregnancies had preterm labor. Iatrogenic preterm delivery ratio was 20% in twin pregnancy and 23% in single pregnancy. In preterm delivery group, only one patient (5.5%) had a previous preterm birth history. We found that preterm delivery group had statistically higher levels of SBA versus term delivery group (p=0.001) (SBAs=100.8 µmol/L versus 29.9 µmol/L) (Table 3). When neonatal outcomes were examined; there was no significant relation between SBA levels and 5^th^ minutes Apgar scores or neonatal birth weights (p=0.353 and p=0.156, respectively).

Nine of 61 patients had the diagnosis of gestational diabetes (14.75%) and another 6 were complicated with preeclampsia (9.8%). The patients with gestational diabetes had the diagnosis of ICP in a mean of 31 weeks. The 6 patients had the diagnosis of preeclampsia after 3 weeks of their diagnosis of ICP (mean 35 weeks). There was not a statistically significant difference in mean values of SBAs between uncomplicated patients and patients complicated with GDM, and preeclampsia (31.6 mmol/L, 43.5 mmol/L, and 51.07 mmol/L, respectively; p=0.669 and p=0.622, respectively).

## DISCUSSION

Although intrahepatic cholestasis of pregnancy is a benign condition and has a mild to moderate derangements to the mother’s health status, it is known to be associated with unfavorable consequences for the pregnancy and the fetus. However, the studies that investigate the correlation between serum bile acids and fetal consequences are inadequate, so far.

In this study, SBA values in patients with preterm delivery were found to be higher, which was statistically significant, when compared to the patients who delivered at term. This finding was compatible with the previous studies^(8,9,13,14,15)^. In a study, frequency of preterm delivery was reported to be 18.7% in patients with ICP, and SBA measurements in those patients were reported to be increased significantly compared to the term parturient^(13)^. But, the latter study enclosed lesser participants than current study. In a cross-sectional study from Sweden, increased frequency of preterm delivery in the previous pregnancies of patients with ICP was established^(15)^. Also, a positive linear correlation between SBA and preterm delivery with every 1-2 mmol/L increase in SBA was asserted in that study. Additionally, authors reported that frequencies of fetal complications, besides preterm delivery, were to be increased particularly when SBA levels were measured over 40 mmol/L. The reported rate of spontaneous preterm delivery was 11%, whereas this ratio was reported as high as 16.7% in patients with ICP in study of Glantz et al.^(15)^. In another Swedish study, 3.3 times increased risk for spontaneous preterm delivery was established in patients with ICP when preterm deliveries between 32^th^ and 37^th^ gestational weeks were evaluated^(8)^. Moreover, patients who were diagnosed to have ICP were found to have 5 times increased risk for iatrogenic preterm delivery, in the same study^(8)^. In this study, 77% of preterm deliveries were spontaneous deliveries among the singleton pregnancies, iatrogenic preterm delivery risk was found not to be increased. Similar to the previous studies, Geenes et al reported that frequency of preterm delivery in singleton pregnancies with severe ICP increased 5.3 times compared to the healthy singletons^(14)^. Contrary to the results of the previously mentioned studies, Rook et al. reported absence of any association between any biochemical marker related to ICP and increased fetal complications^(16)^. In the current study, we found that SBA measurements were not correlated with the weight and APGAR scores of the newborns who were born to mothers with ICP.

The exact mechanism of preterm delivery in patients with intrahepatic cholestasis is not known. It is thought that biliary products increase the sensitivity, and therefore, contractility of the myometrium. Particularly, Germain et al. demonstrated an increased response for oxytocin and increased expression of oxytocin receptors in myometrial tissues incubated with cholic acid^(17)^. Similar to that study, Israel et al demonstrated a similar phenomenon^(18)^. In the latter study, an increased response to the oxytocin was achieved in myometrial cells which were obtained directly from pregnant women with ICP^(18)^.

The other major complication of ICP is intrauterine fetal demise, particularly at term. In the literature, a pregnancy beyond 37^th^ weeks of gestation is addressed as a risk for this unpredicted-, undesired-situation, in a patient with ICP^(8,16,19)^. In the current study, there is no case with antenatal fetal demise. Absence of any fetal loss could be due to close follow-up of the patients and administration of ursodeoxycholic acid (UDCA) treatment where appropriate. The etiology of fetal mortality in patients with ICP is not readily evident. However, there are theories which link this condition to the toxic effects of SBAs^(19,20)^. In a study which was conducted on rats, taurocholic acid-one of the bile acids- was asserted to cause sudden fetal demise by triggering cardiac arrhythmias due to its toxic effects on cardiomyocytes^(21)^. He et al. reported a 29% decrease in volume of placental lobular villous vessels in patients with ICP^(22)^. Placental dysfunction as it is mentioned by He et al. is the other possible cause of intrauterine fetal loss. For this reason, Doppler examination of the fetal umbilical arteries during the follow-up of fetuses in patients with ICP is enounced as a valuable method^(23)^. However, presence of acute anoxia signs rather than chronic anoxia features, presence of correlated fetal weights with the other fetuses at the same gestational age, and presence of normal umbilical artery signs in the examination of fetuses who were lost due to ICP asserted the cause of fetal death due to acute toxic effects of SBA rather than chronic placental insufficiency^(24)^. Therefore, planned preterm deliveries are addressed in the studies to decrease the frequency of intrauterine fetal losses^(25,26)^. Low frequencies of intrauterine fetal demise in this study and other studies in which iatrogenic preterm delivery rates were increased is due to active treatment of the patients.

Lausman et al. did not find increased risk for poor perinatal outcomes in patients with multiple pregnancies complicated by ICP^(27)^. In the current study, there was no statistically significant difference between singleton pregnancies and multiple pregnancies with respect to mean SBAs, frequencies of preterm delivery, and other perinatal morbidities.

Complication rates like postpartum hemorrhage and preeclampsia in patients with ICP were similar to the healthy pregnant women in a retrospective study which comprise 1210 patients^(28)^. But, there are studies that indicate a possible genetic relationship between ICP and preeclampsia^(8,29)^. In this study, the occurrence of preeclampsia among patients with ICP was 9.8%. There was not a statistically significant difference with regard to SMAs between patients with ICP and patients with ICP who were complicated with preeclampsia.

The efficacy of UDCA in treating itching and liver enzyme elevation was shown in plenty of studies^(9,10)^. Additionally, Palma et al. demonstrated efficacy of UDCA in improving fetal results also^(30)^. Furthermore, there are studies, which show that UDCA reduces SBA measurements in fetal chord blood and amniotic fluid, and improves maternal clinical outcome after parturition^(31)^. More studies investigating the favorable effects of UDCA on perinatal outcomes are required.

## CONCLUSION

Patients with ICP constitute a high-risk pregnancy group with respect to fetal and maternal outcomes. Antenatal follow-up of these patients should be performed cautiously for a possible onset of preterm delivery, and scheduled prior to unfavorable fetal conditions occur.

## Figures and Tables

**Table 1:**
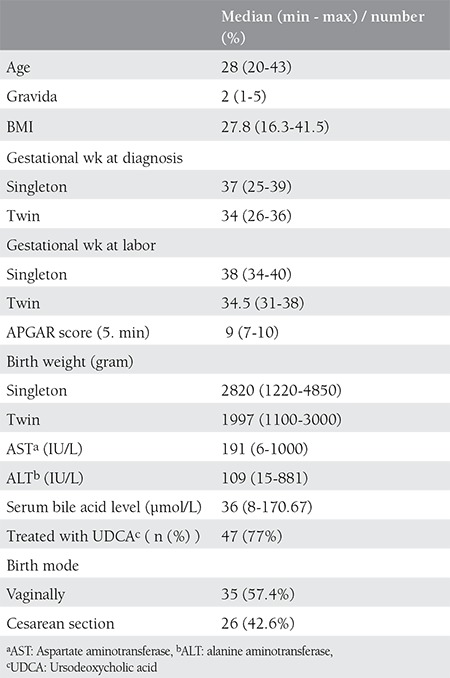
Maternal and Fetal Demographic Values

**Table 2:**
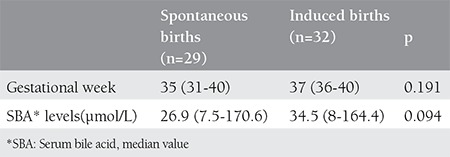
Comparison of spontaneous and induced births of IHC patients

**Table 3:**
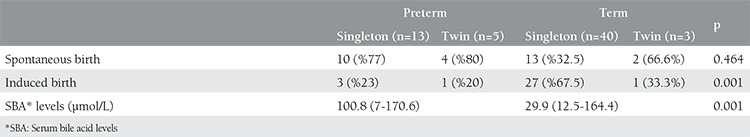
Comparison of preterm and term births of IHC patients
